# Supplementary material for: Effects of trauma-related amputations in children on caregivers: An exploratory descriptive study in a developing country
Source: PLoS One. 2025 Feb 11;20(2):e0313980. doi: 10.1371/journal.pone.0313980 (PMC11813108; doi:10.1371/journal.pone.0313980)
Supplement: S1 File — (DOCX) [file pone.0313980.s001.docx]

**TITLE OF STUDY: EXPERIENCES OF CAREGIVERS OF CHILDREN WITH TRAUMA-RELATED AMPUTATION AT KOFO ANOKYE TEACHING HOSPITAL**

# INTERVIEW GUIDE FOR CAREGIVERS OF CHILDREN WITH

# TRAUMA-RELATED AMPUTATIONS

**Section A: Background Information Form**

1. Age…………………………………
2. Gender: Female [ ]; Male [ ]
3. Place of residence………………………………………………….
4. Nationality ……………………………………………………........
5. Marital status: Married [ ]; Divorced [ ]; Never married [ ]; Separated [ ]
6. Number of children…………………………………………………
7. Occupation …………………………………………………………
8. Level of education …………………………………………………
9. Language(s) spoken …………………………………………………
10. Religion …………………………………………………………….
11. Relationship to child……………………………………..
12. Period of time of caregiving ………………………………………………

**Section B: Guiding Questions**

**Main question:**

**Please share with me your experiences as a caregiver of a child with amputation**

1. Can you please share with me how the experience was from the injury of the child to the amputation process?

Probe:

- What was the mechanism of injury
- Where was your first point of contact to seek for healthcare for your child when the injury occurred?
- What was your reason for your choice of first point of seeking health?
- How was the process in the hospital leading to amputation of the child?

1. Can you please take me through the daily lifestyle and functioning of the child after amputation?

Probe:

- Feeding
- Socialization and hobbies
- Hygiene and Dressing
- Education
- Hospital Reviews and Rehabilitation

1. Please share with me, how has amputation of your child, affected your life as a caregiver.

Probe:

- Daily Life activities
- Social life
- Psychologically
- Finances
- Family Support and Coping strategies

1. Is there any other thing you would like to share with me?

Thank you.
