# Supplementary material for: Effects of trauma-related amputations in children on caregivers: An exploratory descriptive study in a developing country
Source: PLoS One. 2025 Feb 11;20(2):e0313980. doi: 10.1371/journal.pone.0313980 (PMC11813108; doi:10.1371/journal.pone.0313980)
Supplement: S2 File — (DOCX) [file pone.0313980.s002.docx]

| **THEMES** | **SUB -THEMES** | **EXAMPLE QUOTES** |
| --- | --- | --- |
| **Daily Life Activities/ Social Life** | Restricted Movement  Exhaustion | *“…now I’m not able to go anywhere unlike before when he had both legs… my work has been disrupted… there has been changes… my mother passed on yesterday and I was supposed to go but I could not go because of [name of son]”* **(P003_mother of child, lower limb above knee)**  *“...the child is not really that matured, that’s why I’m not able to leave him and go where you have to go because when he needs assistance, someone will have to be there so we always have to be around him.”* **(P006_uncle of child, lower limb above knee)**  *“Oh, he had a sibling who was in school at the time and stopped schooling to cater for him... so our movement has really been affected...”* **(P010_father of child, lower limb above knee)**  *“Because he is young, we are the ones who do everything for him. It’s only recently that he is able to sweep his room”* **(P001_mother of child, lower limb above knee)**  *“I was the one carrying her around all the time for about 4 months...”* **(P002_mother of child, lower limb above knee)**  *“...now if she has to do anything, we have to do it for her. It gets tiring...”* **(P009_father of child, both limbs above knee)** |
| **Finances** | High cost of treatment  **Opportunity cost (Earning loss)** | *“You know when you go to the hospital, everything involves money so we were challenged a bit there... and life in the rural area is not easy. It is even difficult to sell for people to buy so we do not really have money and this also happened.”* **(P006_uncle of child, lower limb above knee)**  *“We have been going to the hospital every 2 days since he was discharged...”*  **(P003_mother of child, lower limb above knee)**  *“...send her to the hospital at Asafo every 3 days and it’s been 10 months now...”* **(P004_uncle of child, lower limb foot)**  *“In terms of finances… the frequent visitation to the hospital has made things difficult a bit because now we go there every month… so the finance is not so good at all.”* **(P002_mother of child, lower limb above knee)**  *“We have really been affected financially… most of our works were affected because I had to be with all the time at Gee* [Komfo Anokye Teaching Hospital] *… we spent everything we had, my wife used to trade, we spent all the capital and went into borrowing… in fact, we are not even done with the loans we took from people.”* **(P010_father of child, lower limb above knee)**  *“Her mother was operating a chop bar so she had some amount of money but since the child got injured, she has been sending her to Asafo and to this place as well so things are a bit difficult for her now.”* **(P004_uncle of child, lower limb foot)**  *“...I am here with my wife and children, my mother had to stop her business at Dunkwa through which she sometimes gave me some support and move here... Secondly, my wife is not able to work because of her condition because there are things that she will need assistance with”* **(P009_father of child, both limbs above knee)** |
| ***Mental Health and Emotions*** | Feeling of guilt and sadness.  Coping Mechanism | *“ I was sad… especially seeing him crawl on his knees when he wakes up and wants to go and urinate… also, when I see his mates play football* [speaks crying]*”* **(P007_mother of child, lower limb above knee)**  *“I used to think that because of this, he has delayed or I have delayed in life …”* **(P010_father of child, lower limb above knee)**  *I’m not able to eat nor sleep, I’m always thinking as to why this problem happened… So, I keep asking why did this happen to me…”* **(P003_mother of child, lower limb above knee**  *“I used to cry a lot… when it happened initially and looking at how my son was just lying down in pain and distress, I could not eat especially the first day they took him to the theatre… I’m not able to eat nor sleep well, I’m always thinking as to why this problem happened… So, I keep asking why did this happen to me…” (P003_mother of child, lower limb above knee)*  *“At first it was tough but other elders also encouraged and advised me and ask me to be grateful because what would I have done if he died. Now even with this condition, both of us are happy even when were alone because the incident has already happened...”* **(P003_mother of child, lower limb above knee)**  *“We encourage ourselves because no matter what, the incident has already happened and I cannot give him a new leg so I have to encourage myself although we did not want that but we encourage ourselves so that it would not happen again. We also talk to the child a lot so that he would not consider any negative thought.”* **(P006_uncle of child, lower limb above knee)** |
