# Supplementary material for: Effects of trauma-related amputations in children on caregivers: An exploratory descriptive study in a developing country
Source: PLoS One. 2025 Feb 11;20(2):e0313980. doi: 10.1371/journal.pone.0313980 (PMC11813108; doi:10.1371/journal.pone.0313980)
Supplement: Attachment — Submitted filename: Response to Reviewers.docx [file pone.0313980.s003.docx]

**RESPONSE TO REVIEWERS**

| **REVIEWER** | **RESPONSE** | **PAGE NUMBER** |
| --- | --- | --- |
| **Reviewer 1** | 1. Reference list has been updated and all changes made throughout the manuscript. Reference number 7 removed and corrected | 1. Page 22 |
| **Reviewer 2** | 1. New Keywords different from words used in the title have been used. 2. Study Setting and Site has been summarized. 3. The last sentence under population was describing the entire population whiles the inclusion criteria was specific under the informal caregivers. The educational status of the caregiver did not influence the effects on the caregiver 4. The differences in the years has been corrected. 5. Repetition of “in-depth interviews” has been corrected. 6. The ages of the children and caregivers has been added to the inclusion criteria. 7. The discussion has been separated into various parts. Additional references have been added in the discussion. | 1.Page 2  2.Page 5  3. Page 5  4. Page 6  5. Page 7  6. Page 6  7.Page 17-20 |
